# GPX5-Enriched Exosomes Improve Sperm Quality and Fertilization Ability

**DOI:** 10.3390/ijms251910569

**Published:** 2024-09-30

**Authors:** Jian Huang, Shuangshuang Li, Yuxuan Yang, Chen Li, Zixi Zuo, Rong Zheng, Jin Chai, Siwen Jiang

**Affiliations:** Key Laboratory of Pig Genetics and Breeding of Ministry of Agriculture, Key Laboratory of Agricultural & Animal Genetics, Breeding and Reproduction of Ministry of Education, Huazhong Agricultural University, Wuhan 430070, China; huangjian978@webmail.hzau.edu.cn (J.H.); lishuang-shuang188@163.com (S.L.); yangyuxuan261@163.com (Y.Y.); lichen14@webmail.hzau.edu.cn (C.L.); z412675858@163.com (Z.Z.); zhengrong@mail.hzau.edu.cn (R.Z.); chaijin@mail.hzau.edu.cn (J.C.)

**Keywords:** exosomes, semen, liquid storage, GPX5

## Abstract

Semen preservation quality affects the artificial insemination success rate, and seminal exosomes are rich in various proteins that are transferable to sperm and conducive to sperm-function preservation during storage. However, the specific effects of these proteins remain unclear. In this study, the specific effects of these proteins on semen preservation quality and fertilization capacity were investigated through a proteomic analysis of seminal exosomes from boars with high conception rates (HCRs) and low conception rates (LCRs). The results revealed significant differences in the expression of 161 proteins between the two groups, with the GPX5 level being significantly higher in the HCR group (*p* < 0.05). The role of GPX5 was further investigated by constructing engineered exosomes enriched with GPX5 (Exo-GPX5), which could successfully transfer GPX5 to sperm. Compared to the control group, Exo-GPX5 could significantly improve sperm motility on storage days 4 and 5 and enhance the acrosome integrity on day 5 (*p* < 0.05). Additionally, Exo-GPX5 increased the total antioxidant capacity (T-AOC) of sperm, reduced the malondialdehyde (MDA) level, and decreased the expression of antioxidant proteins SOD1 and CAT (*p* < 0.05). In simulated fertilization experiments, Exo-GPX5-treated sperm exhibited higher capacitation ability and a significant increase in the acrosome reaction rate (*p* < 0.05). Overall, Exo-GPX5 can improve boar semen quality under 17 °C storage conditions and enhance sperm fertilization capacity.

## 1. Introduction

In modern intensive pig production systems, artificial insemination (AI) is a widely used breeding method, and despite rigorous quality testing of boar semen before AI, conception rates still vary within boar populations [1,2]. AI outcomes can be influenced by numerous factors, with changes in semen quality during storage at 17 °C as a significant influencing factor [3]. As storage time increases, semen is subjected to oxidative stress, leading to a gradual decline in sperm fertility [4,5]. To improve semen storage quality, researchers have primarily focused on the addition of two types of exogenous antioxidants, natural antioxidants [6,7] and synthetic antioxidants [8,9], paying little attention to natural antioxidants from non-plant sources. Note that seminal plasma is rich in various antioxidant substances capable of protecting sperm naturally [10,11], but their specific protective mechanisms require further investigation.

Recent studies have shown that seminal plasma exosomes (SPExos) play a crucial role in maintaining sperm function [10,11]. Mammalian SPExos are characterized by high cholesterol and sphingolipid content, as well as a complex protein composition [12], and these vesicles are primarily produced by the epididymis and prostate [13]. During boar semen storage at ambient temperature, adding seminal exosomes to the diluent can improve sperm quality [14]. Additionally, boar SPExos can also inhibit capacitation-dependent cholesterol efflux in sperm, thereby stabilizing sperm function [13]. Moreover, Murdica et al. found that sperm in vitro could still receive exosomal cargo, benefiting from the transfer of key proteins through exosomes in terms of motility, capability and quality [15]. However, the specific mechanisms by which SPExo proteins contribute to sperm-function preservation remain unclear.

Glutathione peroxidase 5 (GPX5) is an antioxidant enzyme primarily found in the male reproductive system, particularly in the epididymis [16,17]. As a member of the glutathione peroxidase family, the main function of GPX5 is to maintain sperm health and stability by preventing oxidative damage [18]. Chabory et al. found higher rates of natural abortion and developmental defects in the case when GPX5-knockout male mice mated with normal females after one year of age that were associated with oxidative stress damage in sperm [19]. Additionally, GPX5 protein can be transferred to sperm through epididymosomes, which are membrane vesicles secreted by the epididymal epithelial cells that synthesize this enzyme [20]. Moreover, field studies have shown that, during liquid storage at 17 °C, semen with higher GPX5 levels in seminal plasma exhibited a slower decline in sperm motility, enabling these boar populations to have higher farrowing rates and litter sizes [21]. Despite extensive research on the role of GPX5 protein in sperm maturation and in vivo protection, little research has been performed to investigate the specific function and mechanism of GPX5 protein in seminal exosomes during in vitro sperm preservation.

Against the above background, this study aimed to reveal the biological characteristics of seminal exosome proteins by analyzing the proteomic composition of seminal exosomes from boars with different conception rates to screen and identify differentially expressed proteins. Additionally, we explored the role of GPX5 protein in exosomes during sperm liquid storage at 17 °C. Our findings not only facilitate the understanding of the contribution mechanisms of seminal exosomes to sperm function preservation but also offer new strategies for improving boar semen preservation techniques.

## 2. Results

### 2.1. Classification of Boars by Conception Rate

Based on the reproductive information of sows and the semen quality of boars on the farm, six Large White boars were selected and classified into a high conception rate (HCR) and a low conception rate (LCR) group. In Table 1, the HCR group showed a conception rate of 98.44 ± 0.28, significantly higher than the LCR group (89.83 ± 0.44, *p* < 0.05). However, the two groups showed no significant difference in semen quality (*p* > 0.05).

### 2.2. Characterization of Seminal Plasma-Derived Exosomes

**Figure 1:**
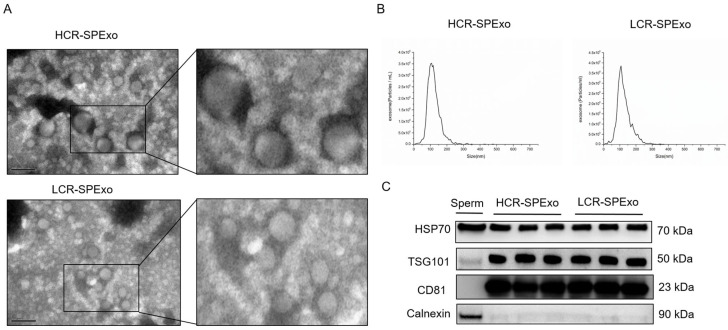
Characterization of boar semen exosomes (SPExo). (**A**) Transmission electron micrographs of semen exosomes from both HCR and LCR groups. Scale bar = 200 nm. (**B**) Representative nanoparticle tracking analysis (NTA) of semen exosomes from both HCR and LCR groups. (**C**) Western blot analysis of seminal plasma exosome marker proteins in the HCR and LCR groups, with sperm cell lysate used as the control group (*n* = 3). HCR, high conception rate; LCR, low conception rate.

The seminal plasma exosomes (SPExos) derived from boars with high and low conception rates were characterized by transmission electron microscopy (TEM), nanoparticle tracking analysis (NTA) and Western blotting. The SPExo from both groups exhibited an intact and cup-shaped morphology (Figure 1A). Their size distribution was characterized by NTA and primarily ranged between 50 nm and 200 nm in the two groups (Figure 1B). The Western blot analysis revealed the presence of three exosomal positive marker proteins (HSP70, TSG101, and CD9) in SPExo samples from both the HCR and LCR groups, while the exosomal negative marker protein Calnexin was not detected in the SPExo samples (Figure 1C). All of these findings showed no significant differences between the two groups in the characterization of seminal exosomes, and the exosomes meet the expected criteria.

### 2.3. Proteomic Analysis of Boar Seminal Plasma Exosomes

**Figure 2:**
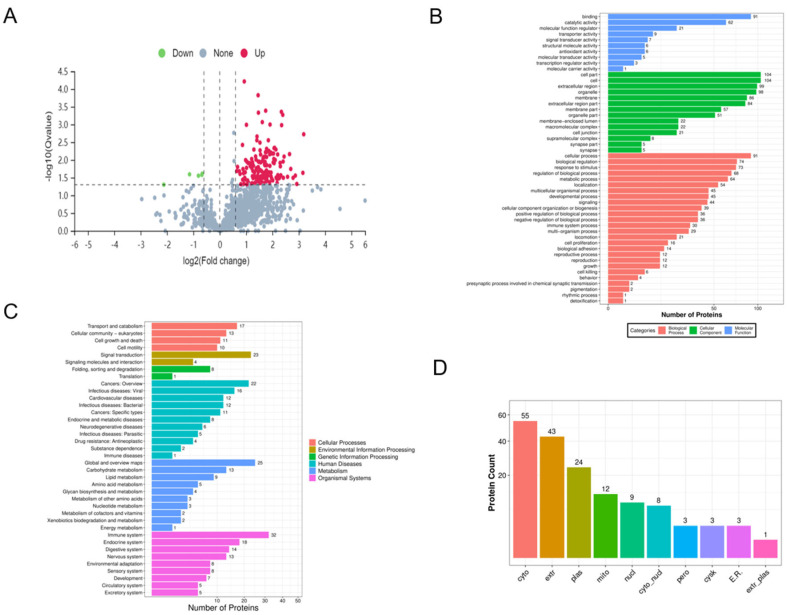
Proteomic analysis of boar semen exosomes from both HCR and LCR groups. (**A**) Volcano plot of differentially expressed exosomal proteins in both HCR (*n* = 3) and LCR groups (*n* = 3). (**B**) GO analysis of differentially expressed exosomal proteins in semen from both HCR and LCR groups. (**C**) KEGG analysis of differentially expressed exosomal proteins in semen from both HCR and LCR groups. (**D**) Cellular component analysis of differentially expressed exosomal proteins in both HCR and LCR groups. HCR, high conception rate; LCR, low conception rate.

Boar seminal exosomes from three HCR and three LCR boars were investigated by proteomic analysis, and proteins detectable in at least one sample were further analyzed, leading to the identification of a total of 1091 proteins. Differentially expressed proteins were identified using a threshold of absolute fold change ≥ 1.3 and *p* < 0.05, and 161 differentially expressed proteins were detected between HCR and LCR groups (Figure 2A). Compared to the LCR group, the HCR group showed a significant upregulation in 156 proteins and a significant downregulation in 5 proteins (the selection of differentially expressed proteins between the two groups is shown in Table 2).

The GO and KEGG analysis results indicated that the upregulated proteins were mainly involved in biological processes, such as the Rap1 signaling pathway, proteoglycans in cancer, glycolysis/gluconeogenesis, and regulation of actin cytoskeleton. Additionally, we found that several differentially expressed proteins were related to the regulation of transport and catalytic processes, such as glutathione peroxidase 5 (GPX5), L-lactate dehydrogenase A chain isoform X3 (LDHA) and acyl-CoA-binding protein (ACBP). The subcellular localization analysis revealed that the differentially expressed proteins had the highest enrichment in the cytoplasm (55 proteins, 34.2%), followed by the extracellular region (43 proteins, 26.7%) and the plasma membrane (24 proteins, 14.9%).

### 2.4. Differential Protein Validation

**Figure 3:**
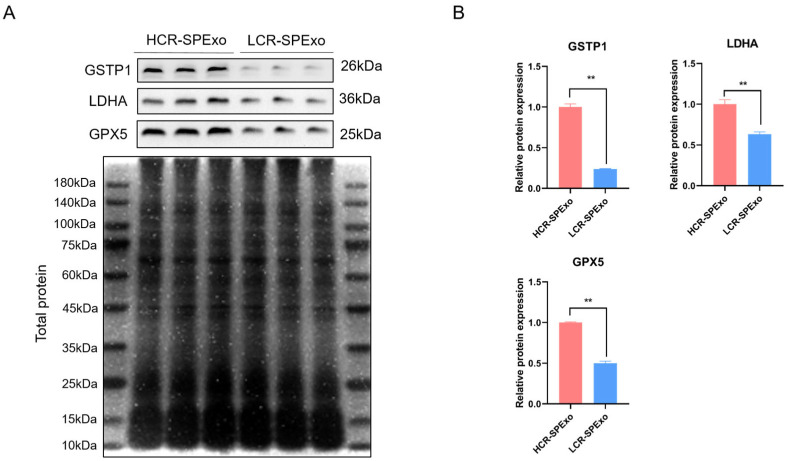
Validation of differentially expressed exosomal proteins by Western blot analysis. (**A**) Proteins of equal mass were separated by electrophoresis, and total proteins were stained with Coomassie Brilliant Blue as a loading control for exosomal proteins. (**B**) Comparison of expression levels of GSTP1, LDHA, and GPX5 in both HCR and LCR groups through densitometric analysis of Western blot bands (*n* = 3). ** *p* < 0.01 and all data are presented as mean ± SEM (*n* = 3). HCR, high conception rate; LCR, low conception rate.

We selected three differentially upregulated proteins (GSTP1, LDHA, and GPX5) for validation of their expression levels by Western blot analysis. The criteria for protein selection included a relatively high expression level, an absolute fold-change cutoff of ≥1.3 between the two groups, and a potential role of these proteins in the sperm fertilization process, as indicated by our GO analysis (based on the UniProt website) or the published literature. Our results confirmed the presence of these proteins in seminal exosomes from both HCR and LCR groups, with lower expression levels detected in LCR group (Figure 3). Among the three proteins, GPX5 plays a crucial role in protecting sperm from oxidative stress damage, which is essential for maintaining reproductive health and fertility, so we focused on the role of GPX5 protein in exosomes in the following experiments.

### 2.5. Construction of GPX5 Protein-Enriched Exosomes

**Figure 4:**
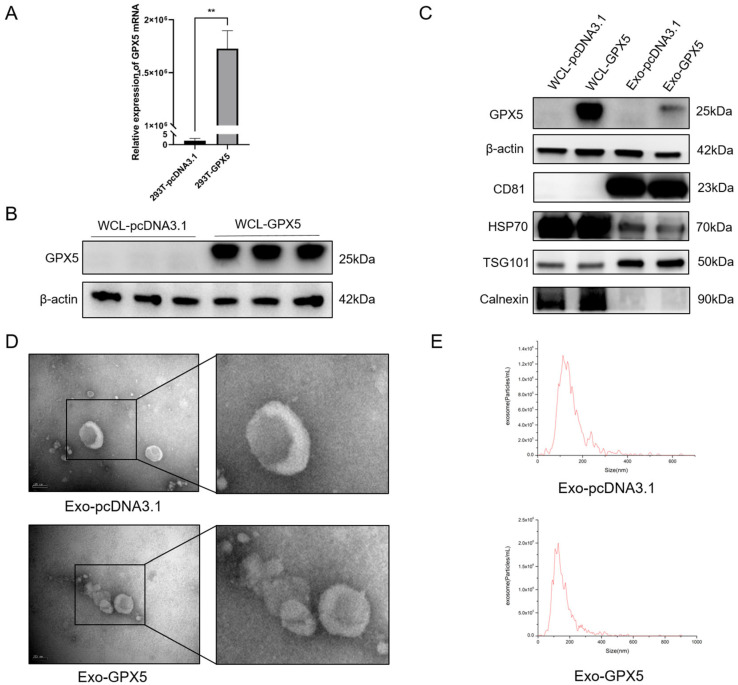
Construction of GPX5-enriched exosomes. (**A**) Expression levels of GPX5 mRNA in 293T cells transfected with an empty pcDNA3.1 vector (designated as 293T-pcDNA3.1) or a pcDNA3.1-GPX5 vector (designated as 293T-GPX5), ** *p* < 0.01. (**B**) Expression levels of GPX5 protein in whole-cell lysates (WCL) of 293T cells transfected with pcDNA3.1 or pcDNA3.1-GPX5 vectors. (**C**) GPX5 expression levels in exosomes (designated as Exo-pcDNA3.1 and Exo-GPX5) isolated from culture supernatants of 293T cells, as determined by Western blot analysis of exosome-positive and -negative markers. (**D**) Transmission electron microscopy (TEM) images of Exo-pcDNA3.1 or Exo-GPX5; scale bar = 100 nm. (**E**) Representative nanoparticle tracking analysis (NTA) of Exo-pcDNA3.1 or Exo-GPX5.

The effect of the differential protein GPX5 in seminal exosomes on sperm quality during semen storage was investigated by using engineered exosomes enriched with GPX5 protein. The 293T cell line is an effective model for constructing engineered exosomes, and by transfecting the porcine GPX5 overexpression vector (pcDNA3.1-GPX5) into 293T cells, we successfully obtained exosomes enriched with GPX5 protein (Exo-GPX5). The mRNA expression level of GPX5 was significantly higher in 293T cells transfected with pcDNA3.1-GPX5 vector versus the empty-vector control group (*p* < 0.01) (Figure 4A). Western blot analysis further demonstrated that the GPX5 protein expression level was markedly increased in the lysates of 293T cells transfected with the pcDNA3.1-GPX5 vector relative to the control group (Figure 4B). Additionally, the exosomes secreted by 293T cells (Exo-GPX5 and Exo-pcDNA3.1) were characterized by Western blotting, TEM, and NTA. Figure 4C shows that, in both the Exo-GPX5 and Exo-pcDNA3.1 groups, the three positive exosome marker proteins (CD81, Hsp70 and TSG101) were expressed, while the negative marker protein (Calnexin) was not detected. The results of TEM and NTA (Figure 4D,E) further demonstrate that the exosomes in both groups exhibited the classic cup-shaped morphology, with similar particle size distributions primarily ranging from 50 to 200 nm.

### 2.6. GPX5 Protein in Exogenous Exo-GPX5 Accumulates in Spermatids

**Figure 5:**
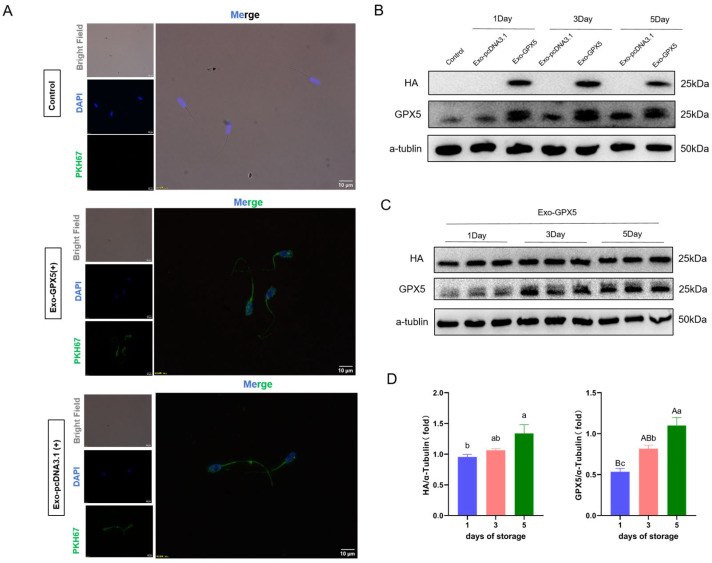
Exo-GPX5 derived from 293T cells can deliver GPX5 protein to sperm cells and accumulate over time. (**A**) Exo-GPX5 and Exo-pcDNA3.1 were labeled with the fluorescent dye PKH67 and excess dye was removed by ultracentrifugation. The supernatant after centrifugation was added to sperm cells as a control group, and PKH67-labeled exosomes were co-incubated with sperm cells for 24 h (PKH67 in green, DAPI in blue); scale bar = 10 µm. (**B**,**C**) After incubating sperm with Exo-GPX5 and Exo-pcDNA3.1 for 5 days, GPX5 and HA protein expression in sperm cells was analyzed by Western blot, with α-tubulin used as the internal control (*n* = 3). (**D**) Expression levels of HA and GPX5 through densitometric analysis of Western blot bands (*n* = 3). Different lowercase letters indicate significant differences at *p* < 0.05, different uppercase letters indicate extremely significant differences at *p* < 0.01 and the same letters indicate no significant differences. All values are expressed as mean ± SEM (*n* = 3).

The exosome-uptake experimental results indicated that both Exo-pcDNA3.1 and Exo-GPX5 (which were labeled with PKH67 fluorescent dye) were successfully taken up by sperm and mainly accumulated in the sperm head and tail midpiece (Figure 5A). Whether the exogenous GPX5 protein was transferred into the sperm was determined by Western blot analysis, and HA protein was only detected in the sperm incubated with Exo-GPX5, but not in the control group or the Exo-pcDNA3.1 group (Figure 5B), confirming that GPX5 was successfully transferred into the sperm through the engineered exosomes. Furthermore, we examined the impact of incubation time on exosome uptake by sperm. With the increase in storage time at 17 °C, the levels of HA and GPX5 proteins in sperm were significantly higher on the fifth day than on the first day (*p* < 0.05). The gradual increase in the levels of both GPX5 and HA proteins in sperm over time indicated that the sperm’s demand for exogenous GPX5 protein is time-dependent during 17 °C storage.

### 2.7. Transferring Exo-GPX5 into Sperm Can Improve Semen Quality

**Figure 6:**
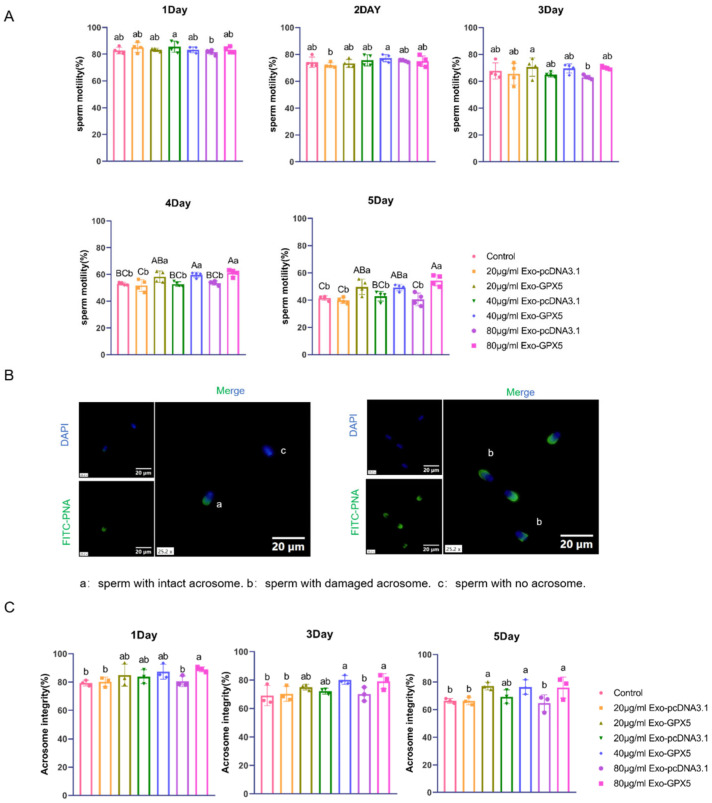
Effects of different concentrations of Exo-GPX5 on sperm quality. (**A**) Sperm motility assessment (*n* = 4). (**B**) Acrosome integrity detection using FITC-PNA and DAPI double staining: (a) sperm with intact acrosomes, (b) sperm with damaged acrosomes, (c) sperm with lost acrosomes; scale bar = 20 μm. (**C**) Analysis of sperm acrosome integrity rate (*n* = 3); different lowercase letters indicate significant differences at *p* < 0.05, different uppercase letters indicate extremely significant differences at *p* < 0.01 and the same letters indicate no significant differences. All values are expressed as mean ± SEM.

Sperm motility is a key indicator of semen quality. In this study, sperm motility was measured after adding different concentrations of Exo-GPX5 or control exosomes (Exo-pcDNA3.1) separately to dilute boar semen samples stored at 17 °C (Figure 6A). The results showed that the sperm motility gradually decreased with increasing storage time. On days 1, 2 and 3 of storage, there was no significant difference in sperm motility between the various concentrations of the Exo-GPX5 groups and the control group (*p* > 0.05). However, on days 4 and 5, sperm motility was significantly higher in the 20, 40 and 80 μg/mL Exo-GPX5 groups than in the control group (*p* < 0.01). Acrosome integrity is also a crucial indicator for sperm quality and fertilization capacity, so we assessed the impact of different concentrations of Exo-GPX5 and Exo-pcDNA3.1 on the acrosome integrity of sperm stored at 17 °C (Figure 6B,C). Similar to sperm motility, acrosome integrity gradually decreased with increasing storage time. On storage days 1 and 3, the acrosome integrity was significantly higher in the 80 μg/mL Exo-GPX5 group than in the control group (*p* < 0.05). By day 5, acrosome integrity was significantly higher in the 20 and 80 μg/mL Exo-GPX5 groups than in the control groups (*p* < 0.05). These findings suggest that, even at the lowest concentration of 20 μg/mL, Exo-GPX5 can effectively improve sperm motility and acrosome integrity during 17 °C storage, so we further explored the effect of 20 μg/mL Exo-GPX5 on sperm antioxidant capacity and fertilization ability in the subsequent experiments.

### 2.8. Transferring Exo-GPX5 to Sperm Enhances Sperm Antioxidant Capacity

**Figure 7:**
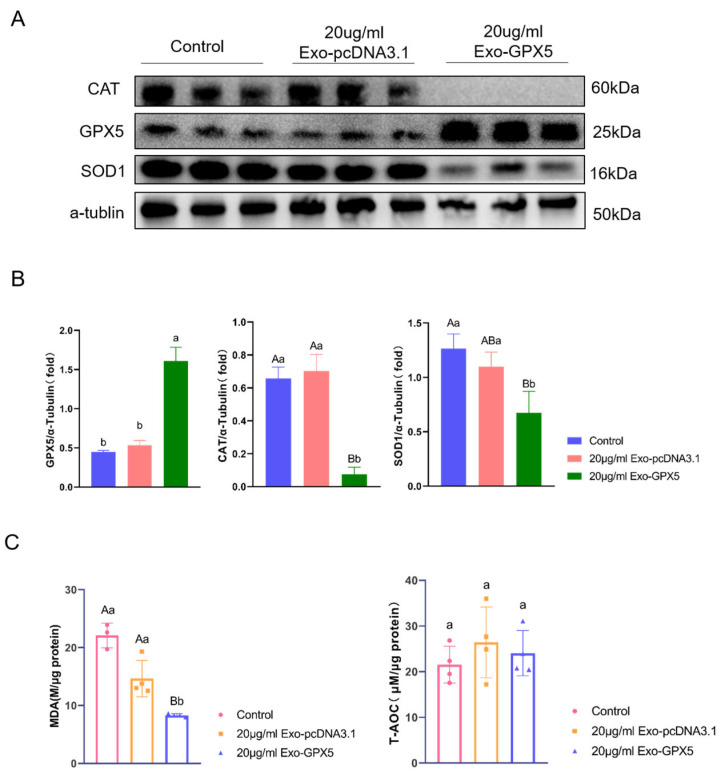
Effects of 20 μg/mL Exo-GPX5 on the antioxidant levels of boar sperm stored at 17 °C. (**A**) Levels of antioxidant-related proteins (CAT, GPX5, and SOD1) in sperm, with α-tubulin as the internal control (*n* = 3). (**B**) Densitometric analysis of Western blot bands (*n* = 3). (**C**) The effect of 20 μg/mL Exo-GPX5 on MDA levels and T-AOC activity (*n* = 4). Different lowercase letters indicate significant differences at *p* < 0.05, different uppercase letters indicate extremely significant differences at *p* < 0.01 and the same letters indicate no significant differences. All values are expressed as mean ± SEM.

GPX5 is a crucial antioxidant protein for protecting sperm, so we evaluated the effect of adding 20 μg/mL Exo-GPX5 on sperm antioxidant capacity during storage. The levels of antioxidant-related proteins in sperm on storage day 5 at 17 °C are shown in Figure 7A,B. Compared to the control and empty vector groups, the Exo-GPX5 group showed significant enhancement in the expression of GPX5 (*p* < 0.05), in contrast to significant reduction in the expression of antioxidant-related proteins SOD1 and CAT in the control and empty vector groups (*p* < 0.01). Additionally, the antioxidant capacity of sperm was further assessed using MDA and T-AOC assay kits (Figure 7C) and compared with the control group, the Exo-GPX5 group showed a significantly lower MDA level (*p* < 0.01) and a slightly but not significantly higher T-AOC level. These findings confirmed that 20 μg/mL Exo-GPX5 can effectively enhance the antioxidant capacity of sperm during in vitro storage.

### 2.9. Transferring Exo-GPX5 into Sperm Improves Sperm Fertilization Ability

**Figure 8:**
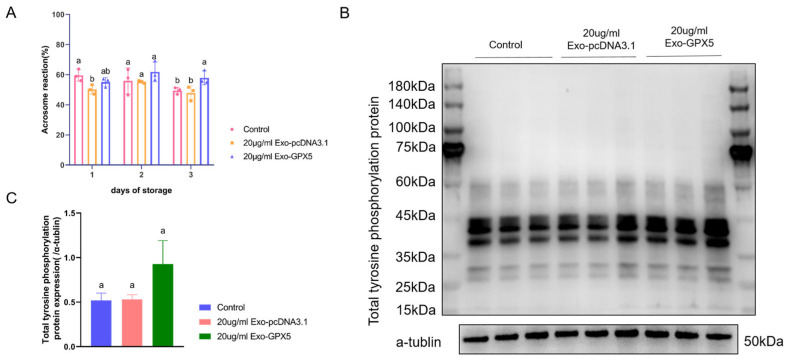
Sperm fertilization ability is enhanced by 20 μg/mL Exo-GPX5. (**A**) Acrosome reaction rate in sperm. (**B**) Expression of tyrosine-phosphorylated proteins in sperm, with α-tubulin as the internal control. (**C**) Densitometric analysis of Western blot bands (*n* = 3). Different lowercase letters indicate significant differences at *p* < 0.05 and the same letters indicate no significant differences. All values are expressed as mean ± SEM (*n* = 3).

Under capacitation conditions, boar sperm was incubated in vitro at 37 °C for 2 h, followed by the addition of exogenous progesterone to simulate the female reproductive tract environment, inducing sperm capacitation and the acrosome reaction. As reflected in Figure 8A, no significant difference was observed in the acrosome reaction rates between the Exo-GPX5 group, the empty vector group and the control group on days 1 and 3 (*p* > 0.05). However, on day 5, the acrosome reaction rate in the Exo-GPX5 group was significantly higher than in the control group (*p* < 0.05). The tyrosine phosphorylation level in sperm is an important indicator of capacitation ability, so we assessed the tyrosine phosphorylation level in sperm after capacitation on storage day 5. The results showed that the addition of 20 μg/mL Exo-GPX5 increased tyrosine phosphorylation levels in sperm (Figure 8B,C). These findings suggest that Exo-GPX5 can enhance the fertilization ability of sperm during storage, particularly as storage time increases.

## 3. Discussion

Seminal plasma exosomes play a critical role in semen preservation by effectively protecting sperm from oxidative-stress damage, inhibiting premature capacitation and acrosome reaction, and maintaining sperm motility and fertilization ability [13,14]. However, current research on seminal exosomes has primarily focused on the effects of exosomes on sperm phenotypes [14,15] or the compositional differences in exosomes [22,23,24], and less research efforts are devoted to the role of specific components within exosomes in these phenotypes. In the present study, we focused particularly on the GPX5 protein by proteomic composition analysis of seminal exosomes, and GPX5 was significantly upregulated in the high-conception-rate group. GPX5 is an epididymis-specific antioxidant enzyme and mainly expressed in the head of epididymis [25,26] to regulate the concentration of reactive oxygen species (ROS) [19]. GPX5 can effectively prevent sperm lipid peroxidation by binding to sperm in epididymis to maintain the integrity and stability of sperm DNA [27] and preventing premature acrosome reactions in the tail of epididymis to ensure proper sperm maturation [28]. Currently, the role of GPX5 in exosomes during semen storage has not been extensively studied and our findings suggest that, when delivered to sperm via exosomes, GPX5 protein could help maintain the boar semen quality during 17 °C storage.

Due to its minimal expression of endogenous receptors required for extracellular ligands and high transfection efficiency, the 293T cell line is commonly used for exogenous gene expression studies and is suitable for constructing engineered exosomes enriched with specific proteins [29]. Vilanova-Perez et al. demonstrated that exosomes derived from HEK293T cells do not adversely affect the structure or function of boar sperm when applied in vitro [30]. Inspired by this, we constructed a porcine GPX5 overexpression vector and transfected it into 293T cells to obtain GPX5-enriched exosomes. We found that exogenous GPX5 protein could be delivered into sperm through exosomes during storage and accumulate over storage time. A possible explanation for this accumulation is that, as semen is stored in vitro, the antioxidant defense system in semen gradually becomes compromised over time [31], because sperm has limited antioxidant defense capacity to counteract ROS attacks, making seminal plasma a source of antioxidants to keep ROS levels within physiological ranges [32,33]. In Taylor’s study [20], GPX5 was shown to bind to sperm membranes in vivo through epididymosomes rather than directly from the soluble portion of the epididymal fluid. This suggests that, as storage time increases, GPX5 protein in seminal exosomes may bind to sperm during storage, thereby enhancing their antioxidant capacity. However, the specific binding mechanism and mode between exosomes and sperm require further investigation.

This study classified boars based on their conception rates and found no significant differences in semen quality (including motility, density, and volume) between the HCR and LCR groups (*p* > 0.05). However, there was a significant difference in conception rates (*p* < 0.05). It is important to note that the semen quality parameters for both groups were measured on the day of collection, while the semen used for artificial insemination is typically stored at 17 °C for 1–5 days before being used for sow breeding. Research indicates that storing semen at 17 °C leads to oxidative stress, resulting in decreased sperm motility and an increased proportion of sperm that undergo premature capacitation and acrosome reaction, which may reduce the fertilizing ability of boars [11,34]. Among the semen exosome proteins in the HCR group, the significantly upregulated GPX5 protein may play a role in sperm protection in this process. In the present study, adding Exo-GPX5 to semen was shown to improve sperm motility and acrosome integrity during storage, but the effect on sperm motility was not significant during the first three days of storage, possibly because oxidative stress levels in semen are relatively low in the early storage stages. As storage time increases, the balance is disrupted between ROS production and scavenging capacity in sperm, leading to excessive ROS production. Due to their relatively high content in the phospholipids of porcine sperm membranes, polyunsaturated fatty acids are particularly susceptible to oxidative damage [35,36]. One of the by-products of lipid peroxidation is MDA, an important indicator of oxidative stress in sperm, while T-AOC represents the overall antioxidant capacity of sperm. Our results showed that adding Exo-GPX5 could significantly reduce MDA levels in sperm while increasing T-AOC values, suggesting that Exo-GPX5 could contribute to scavenging free radicals, thus inhibiting excessive ROS production and protecting sperm. However, we also found that Exo-GPX5 addition led to a decrease in the expression of antioxidant-related proteins CAT and SOD1 in sperm, which may be related to the dynamic balance of antioxidant proteins in sperm, and the gradual accumulation of GPX5 may lead to a decrease in the expression of other antioxidant proteins to maintain overall redox balance. In in vivo simulated fertilization experiments, we found that, on storage day 5, the 20 μg/mL Exo-GPX5 group showed enhancement in sperm capacitation ability and acrosome reaction rate. Barranco et al. also indicated that boars with a higher total antioxidant capacity in their seminal plasma had significantly higher fertilization rates than those with a lower total antioxidant capacity [37]. This indicated that adding Exo-GPX5 to semen stored in liquid form can maintain T-AOC levels and inhibit excessive MDA production through the antioxidant action of GPX5, thereby improving semen quality and fertilization capacity.

In this study, we demonstrated the importance of engineered exosomes (Exo-GPX5) in maintaining sperm motility, acrosome integrity, antioxidant capacity, and enhancing fertilization ability during liquid storage. Currently, exogenous antioxidants are commonly used in semen preservation to improve storage quality. Exo-GPX5, as a cell-derived antioxidant additive, offers a new approach for improving boar semen preservation quality. Given the potential heterogeneity in exosomes from different sources and the main production of seminal exosomes by epididymis and prostate, future research can focus on the development of engineered exosomes derived from reproductive system cells, such as those from epididymis and prostate, because exploring the efficiency of these reproductive system-derived exosomes in delivering GPX5 protein or other specific proteins and evaluating their performance in semen preservation are of significant research value.

## 4. Materials and Methods

### 4.1. Semen Sample Collection

This study utilized the production data from a large pig farm in Northern China. Descriptive statistics were used to organize and analyze the boar semen data and the reproductive information of the sows. The breeding study was conducted using the same breed boars and sows for consistency, with the most commonly raised breed on the farm (Large White pigs) as the subject. Sows were inseminated twice by AI to ensure the same semen was used for both inseminations. The accuracy of reproductive data was ensured by only including the data from the first estrus of the sows. Based on the conception rates, six Large White boars were selected for semen collection and categorized into HCR and LCR groups. The breeding information for boars and sows can be found in Supplementary Table S1. All the six boars were sexually mature and semen samples were collected from each individual using a gloved-hand method. The sperm-rich portion of the semen was obtained from each boar, and sperm motility was assessed using the CASA system (IVOS II, France). Semen samples were centrifuged (800× *g*, 17 °C, 15 min) to separate the sperm from the supernatant, followed by exosome extraction from the supernatant.

### 4.2. Isolation of Seminal Plasma Exosomes

Isolate pig seminal plasma exosomes (SPExos) using ultracentrifugation according to previously described methods [14,38]. For seminal plasma, centrifuge at 3000× *g* for 10 min at 4 °C to remove intact and dead sperm cells. Centrifuge the supernatant at 10,000× *g* for 30 min at 4 °C to remove cell debris and small fragments of undissolved semen gel. Next, centrifuge the supernatant at 20,000× *g* for 30 min at 4 °C to remove microvesicles. Filter the resulting supernatant through a 0.22 μm filter (Millipore, Burlington, MA, USA) to remove bacteria and impurities. Transfer the filtrate to ultracentrifuge tubes (Beckman, Brea, CA, USA) and subject it to ultracentrifugation at 120,000× *g* for 2 h at 4 °C using an ultracentrifuge (Beckman Optima XE-90). Resuspend the pellet in PBS (filtered through a 0.22 μm filter) (Gibco, New York, NY, USA) and store at −80 °C until use.

### 4.3. Transmission Electron Microscopy

The morphology of SPExo was observed by TEM, as previously reported [29]. A 10 μL aliquot of diluted exosome suspension was placed on a membrane, followed by inverting a sample-bearing copper grid onto the membrane and incubation at room temperature for 20 min. Next, tweezers were used to pick up the edge of the copper grid, followed by blotting the liquid from the side of the grid using electron microscopy-grade filter paper, and then adding 1% uranyl acetate staining solution to the grid. After incubation in the dark for 1–3 min, the staining solution was blotted from the side with filter paper. Subsequently, the grid was air-dried at room temperature for 10–20 min, and the samples were observed under a 100 kV transmission electron microscope (H-7650, HITACHI, Tokyo, Japan) at the National Key Laboratory of Huazhong Agricultural University.

### 4.4. Nanoparticle Tracking Analysis

The size and concentration of seminal plasma exosomes were analyzed using the ZetaView system (Particle Metrix, Meerbusch, Germany). NTA measurements were recorded and analyzed at 11 positions. The ZetaView system was calibrated using 110 nm polystyrene particles.

### 4.5. Western Blotting

Protein concentrations were determined using the BCA protein assay kit, as instructed by the manufacturer (A55864, Thermo Scientific, Waltham, MA, USA). Proteins were electrophoretically separated using 10% SDS-PAGE gels and then transferred to PVDF membranes. After blocking with a rapid blocking buffer (PS108P, Epizyme Biotech, Shanghai, China), the membranes were incubated first with primary antibodies overnight at 4 °C, and then with secondary antibodies (Proteintech, Rosemont, IL, USA), followed by detection using an enhanced chemiluminescence (ECL) system. The proteins were visualized using an enhanced chemiluminescence reagent. The antibodies used in this study are listed in Table 3. In this study, we utilized electrophoresis to separate equal amounts of exosomal proteins and stained the total exosomal proteins with Coomassie Brilliant Blue, serving as a reference for exosomal protein quantification. For cellular samples, we used β-actin as the reference protein, while α-tubulin was selected as the reference for sperm samples. Relative protein expression levels were investigated via a grayscale analysis using ImageJ 1.54K software (National Institutes of Health, Bethesda, MD, USA).

### 4.6. Proteomic Analysis

High coverage, precision, and reproducibility in protein quantification can be achieved by the next-generation label-free quantitative proteomics technology through the Data-Independent Acquisition (DIA) mode. The DIA process includes spectral library construction, DIA data acquisition, and data analysis. First, a spectral library was constructed by collecting high-quality peptide information from samples as a template for subsequent data analysis. Then, in DIA mode, large numbers of sample data were acquired, with the mass spectrometer cycling through 64 variable wide precursor ion selection windows and simultaneously fragmenting multiple peptides, ensuring high reproducibility in data acquisition. Finally, the spectral library was used for deconvolution of DIA data to obtain qualitative and quantitative information on peptides and proteins through differential analysis and biological function analysis, using the MSstats package (https://www.bioconductor.org/packages/release/bioc/html/MSstats.html, accessed on 1 September 2024). For each sample, 100 μg of protein solution was digested with trypsin at a protein ratio of 40:1 for 4 h, followed by desalting using Strata X columns and vacuum drying. The peptide samples were reconstituted in mobile phase A, centrifuged and injected into the Shimadzu LC-20AD liquid chromatography system with a Gemini C18 column for separation, gradient elution and elution-peak monitoring. Finally, the samples entered the trap column for enrichment and desalination and then separated on a self-packed C18 column (75 µm inner diameter, 3 µm column particle size and 25 cm column length). The mass spectral data were analyzed using Thermo Proteome Discoverer 2.1 software by searching against the Bos Taurus Uniprot database (http://www.uniprot.org/proteomes/UP000009136, accessed on 16 September 2021). Gene ontology (GO) analysis, including molecular function (MF), cellular component (CC), cellular component (CC) and biological process (BP), was performed using the DAVID online tool. Pathway analysis was conducted using the KEGG database (https://www.kegg.jp/, accessed on 16 September 2021).

### 4.7. Construction of GPX5-Enriched Exosomes

We constructed a porcine GPX5 overexpression vector (pcDNA3.1-GPX5), which carries an HA tag (TACCCATACGACGTACCAGATTACGCT) following the GPX5 sequence. Transfect the 293T cell line with pcDNA3.1-GPX5 and the empty vector (pcDNA3.1). After transfection for 48 h, the supernatant was discarded, and the cells were subjected to serum-free medium starvation for 48 h. We then collected the supernatant and extracted exosomes from the cell supernatant using the same method as for seminal plasma exosomes. The expression level of GPX5 in the constructed 293T cells was detected by quantitative RT-PCR and the protein levels of GPX5 in the transfected 293T cells and their exosomes were detected and analyzed by Western blotting. We gave the exosomes enriched with porcine GPX5 protein the name Exo-GPX5 and the exosomes obtained from 293T cells transfected with the empty vector were named Exo-pcDNA3.1.

### 4.8. Sperm Uptake of Exosomes

To analyze sperm uptake of exosomes, Exo-GPX5 and Exo-pcDNA3.1 were labeled with the green membrane dye PKH67 (MINI67-1KT, Sigma-Aldrich, St. Louis, MO, USA). Next, excess dye was removed from the exosomes by ultracentrifugation at 120,000× *g* and 4 °C for 2 h to obtain fluorescent exosomes. The supernatant after ultracentrifugation was added to sperm as a negative control. Subsequently, boar sperm was incubated with fluorescent exosomes (final concentration: 40 µg/mL) at 17 °C for 24 h, followed by fixation with 4% paraformaldehyde, staining with DAPI and observing the fluorescence signals under a fluorescence inverted microscope (BX53; Olympus, Shinjuku, Japan). Meanwhile, fluorescently labeled Exo-GPX5 and Exo-pcDNA3.1 were incubated with sperm for five days and sperm samples were collected on days 1, 3 and 5. The expression levels of HA protein and GPX5 protein in the sperm were detected by Western blotting.

### 4.9. Semen Storage at 17 °C: Experimental Design

This study includes two experiments. In the first experiment, different concentrations of Exo-GPX5 and Exo-pcDNA3.1 (0, 20, 40, and 80 μg/mL) were added to boar semen stored at 17 °C, and sperm motility and acrosome integrity were assessed from day 1 to day 5 to determine the minimum concentration required to enhance sperm motility and acrosome integrity. In the second experiment, using the minimum concentration determined from the first experiment, changes in the levels of relevant antioxidant proteins (GPX5, CAT and SOD1) in sperm stored for 5 days were analyzed, along with the antioxidant levels in sperm using T-AOC and MDA assay kits. Additionally, sperm acrosome reaction rate and in vitro capacitation ability were evaluated on days 1, 3 and 5 under simulated in vivo fertilization conditions.

### 4.10. Sperm Motility and Acrosome Integrity Assessment

Sperm motility was measured using a semen analyzer (SQA-6100vet, Xiangxin, Shanghai, China). Briefly, 2 μL of semen sample was placed on a specialized counting chamber, and sperm motility was automatically assessed by the system. Acrosome reaction or damaged-sperm percentages were evaluated using FITC-PNA conjugated with fluorescein isothiocyanate (FITC-PNA) and 4′,6-diamidino-2-phenylindole (DAPI) double staining. Briefly, sperm samples were smeared on glass slides, air-dried, and fixed with methanol at 37 °C for 15 min. After three washes with phosphate-buffered saline (PBS), the samples were incubated with FITC-PNA (10 μg/mL) and DAPI (6 μg/mL) at 37 °C in the dark for 30 min. Finally, slides were mounted with fluorescence mounting medium (#S3023; Dako, Carpinteria, CA, USA) and examined under a fluorescence upright optical microscope (Nikon Eclipse E200). The acrosomal status was observed and 200 sperm cells per sample were randomly counted. Sperm with intact acrosomes exhibited a smooth, uniform and hemispherical fluorescence at the edge of the sperm head, while sperm with damaged acrosomes showed irregular edges, acrosomal loss, or swelling.

### 4.11. In Vitro Capacitation and Progesterone-Induced Acrosome Reaction

On days 1, 3 and 5 during 17 °C storage, sperm samples were collected separately and centrifuged at 800× *g* and 17° C for 10 min, and then the resulting pellet was resuspended in BWW medium (G2585, Solarbio, Beijing, China) for capacitation. The acrosome reaction was induced as described previously; the sperm samples were incubated at 37 °C for 2 h, followed by adding progesterone (final concentration: 10 µg/mL) to induce the acrosome reaction through further incubation for 30 min for acrosome reaction estimation [10,30]. The acrosome reaction rate (%) was calculated as 1—acrosome integrity rate (%). Protein tyrosine phosphorylation is a key marker of sperm capacitation and changes in sperm tyrosine phosphorylation levels were analyzed by Western blotting to assess sperm capacitation ability.

### 4.12. Evaluation of T-AOC and MDA Content

To assess the antioxidant capacity of sperm stored at 17 °C for five days, T-AOC was measured using a total antioxidant capacity assay kit (S0121, Beyotime, Shanghai, China), and MDA content was measured using a lipid peroxidation assay kit as instructed by the manufacturer (S0131S, Beyotime, Shanghai, China). Briefly, sperm extracts were prepared by sonication (operating at 25%, 3 s on, 5 s off, 5 cycles) in ice-cold buffer (50 mM Tris-HCl [pH 7.5], 5 mM EDTA and 1 mM DTT). Sperm protein concentration was determined using the BCA protein assay kit (A55864, Thermo Scientific, Waltham, MA, USA). For T-AOC analysis, the supernatant was collected and mixed with the T-AOC reaction buffer, and T-AOC in the semen samples was expressed as M/μg protein. For MDA analysis, cell lysates were centrifuged (12,000× *g*, 10 min) to remove debris, followed by collecting the supernatant and mixing it with the MDA reaction buffer. MDA content in sperm samples was expressed as nmol/μg protein.

### 4.13. Statistical Analysis

All analyses were performed using Statistical Product and Service Solutions (SPSS 22 for Windows; SPSS, Chicago, IL, USA). Experimental results were expressed as mean ± standard error of the mean (SEM). A *t*-test was used for comparison between two groups, while multiple comparisons were conducted using one-way analysis of variance (ANOVA), followed by post hoc pairwise comparisons using the Tukey test. Statistical significance was set at *p* < 0.05.

## Figures and Tables

**Table 1:** Classification of boars by conception rate and semen quality information.

Parameter	HCR (*n* = 3)	LCR (*n* = 3)	*p*-Value
Conception rate (%)	98.44 ± 0.28	89.83 ± 0.44	<0.01
Number of matings (n)	89.33 ± 20.85	77.00 ± 27.30	0.738
Sperm motility (%)	81.48 ± 1.28	79.34 ± 1.91	0.404
Semen volume (mL)	245.88 ± 38.32	230.34 ± 33.79	0.776
Sperm density (10^8^/mL)	3.37 ± 0.09	2.97 ± 0.15	0.084

All values are shown as mean ± SEM (*n* = 3).

**Table 2:** Significant differences between HRC and LRC groups in protein expression of semen exosomes.

Protein Accession	Protein Name	Fold of Change (HCR/LCR)	*p*-Value
O18994	Glutathione peroxidase 5	4.14	0.0094
O62787	Glucose transporter type 3	3.46	0.0141
O97763	Niemann Pick type C2 protein homolog	2.01	0.0010
P00336	L-lactate dehydrogenase B chain	4.50	0.0340
P00339	L-lactate dehydrogenase A chain isoform X3	2.62	0.0414
P00571	Adenylate kinase isoenzyme 1	5.24	0.0005
P08132	Annexin A4	2.71	0.0286
P10668	Cofilin-1	3.73	0.0069
P12026	Acyl-CoA-binding protein	3.34	0.0060
P12309	Glutaredoxin-1	3.41	0.0060
P20735	Gamma-glutamyltranspeptidase 1	2.66	0.0022
P26042	Moesin	4.89	0.0121
P46410	Glutamine synthetase	5.31	0.0319
P50578	Aldo-keto reductase family 1 member A1	3.41	0.0222
P80310	Protein S100-A12	5.94	0.0299
P80928	Macrophage migration inhibitory factor	2.93	0.0117
P82460	Thioredoxin	2.36	0.0116
Q07717	Beta-2-microglobulin precursor	1.60	0.0157
Q29549	Clusterin precursor	3.23	0.0453
Q2PKF4	Guanine nucleotide-binding protein G(q) subunit alpha	2.97	0.0448
Q45FY6	Hypoxanthine-guanine phosphoribosyltransferase	2.75	0.0327
Q6QAQ1	Actin	3.94	0.0066
Q8SPJ0	Inactive ribonuclease-like protein 10 precursor	5.43	0.0080
Q8WNR0	High-affinity copper uptake protein 1	2.58	0.0497

**Table 3:** Western blot antibodies.

Antibody	Information
anti-HSP70	T55496F, 1:1000; Abmart (Berkeley Heights, NJ 07922, USA)
anti-TSG101	A1692, 1:1000; ABclonal (Woburn, MA, USA)
anti-GSTP1	PK09035S, 1:500; Abmart
anti-LDHA	PK37055S, 1:1000; Abmart
anti-GPX5	PK99434S, 1:1000; Abmart
anti-β-actin	AC026, 1:1000; ABclonal
anti-CD81	A4863, 1:1000; ABclonal
Calnexin	A4846, 1:1000; ABclonal
anti- HA	M20003S, 1:5000; Abmart
anti-alpha Tubulin	T40103S, 1:5000; Abmart
anti-CAT	R23734, 1:1000; Zenbio (Durham, NC, USA)
anti-SOD1	R25829, 1:1000; Zenbio
anti-Phosphotyrosine	sc-7020, 1:500; Santa Cruz Biotechnology, Inc. (Dallas, TX, USA)

## Data Availability

The data supporting the findings of this study are available from the first author upon reasonable request.

## Supplementary Materials

The following supporting information can be downloaded at: https://www.mdpi.com/article/10.3390/ijms251910569/s1.
